# Measurement of Revascularization Effect Using Near Infrared Spectroscopy in Below the Knee Arteries

**DOI:** 10.31083/j.rcm2309299

**Published:** 2022-09-05

**Authors:** Tomas Baltrūnas, Gabija Pikturnaitė, Austėja Račytė, Vaida Baltrūnienė, Valerija Mosenko, Arminas Skrebūnas, Gediminas Vaitėnas, Stasys Ščerbinskas, Sigitas Urbonavičius, Kęstutis Ručinskas

**Affiliations:** ^1^Faculty of Medicine, Vilnius University, LT-03101 Vilnius, Lithuania; ^2^Vilniaus Miesto Klinikinė Hospital, LT-10207 Vilnius, Lithuania; ^3^Department of Vascular Surgery, Zealand University Hospital, DK-4000 Roskilde, Denmark

**Keywords:** near-infrared spectroscopy, blood perfusion, wound healing, chronic limb-threatening ischemia, below the knee, chronic total occlusion

## Abstract

**Background:**

Current methods evaluating tissue ischemia are based mainly on evaluating blood flow and not tissue perfusion. However, diabetes mainly affects small vessels and blood flow evaluation is insufficient. The aim of the trial was to evaluate the feasibility of NIRS in measuring perfusion changes during chronic total occlusion (CTO) revascularization in below the knee (BTK) arteries.

**Methods:**

A prospective observational study was performed. During the endovascular revascularization procedure, tissue oxygenation changes were measured using three NIRS sensors. Postoperative angiographies and 30 days wound healing was evaluated.

**Results:**

The study enrolled 30 patients with chronic limb threatening ischemia, occluded below the knee arteries, Rutherford 5. Mean age 74.7 ± 11.2 years, 16 (53%) of the patients had diabetes mellitus, 10 (33%) had end-stage renal disease. A statistically significant NIRS ${\mathrm{rSO}}_{2}$ increase was observed on sensors near the wound after the revascularization, *p* = 0.001. Thirty days follow-up visits included 27 patients, because 3 patients had died. Comparing good wound healing group with poor wound healing group intraoperative NIRS ${\mathrm{rSO}}_{2}$ increase difference was statistically significant, *p* = 0.017.

**Conclusions:**

The study confirmed tissue perfusion increase could be detected using NIRS during revascularization of below the knee arteries. An intraoperative increase of NIRS ${\mathrm{rSO}}_{2}$ proved to predict wound healing results.

Type of Research: Single center prospective observational study.

Key Findings: Tissue perfusion increase was detected using NIRS during revascularization of below the knee arteries in 30 patients. NIRS was superior in predicting wound healing compared to a blinded angiography evaluation.

Take home Message: Tissue perfusion measurement is of increasing importance due to growing incidence of small vessel disease, which are resulted by diabetes and end stage renal disease. However, there is no validated method to evaluate it. NIRS showed promising intraoperative monitoring results in a very restricted cohort.

Summary: NIRS is feasible method for detecting tissue perfusion changes during endovascular revascularization of BTK and BTA arteries. This proof of concept does not translate into clinical practice with existing devices in the market.

## 1. Introduction

Chronic limb-threatening ischemia (CLTI) is an end-stage of peripheral artery disease (PAD), which includes a broad and heterogeneous group of patients [1]. Because of the aging society and the increasing prevalence of diabetes mellitus, the altered vascular bed shifts from aortoiliac to below the knee (BTK) and below the ankle (BTA) [2]. The new term “small artery disease” (SAD) is coming to stage not as a subgroup of PAD but more as an independent disease caused by medial arterial calcification [3, 4]. This increases the importance of blood perfusion measurement.

Current methods evaluating tissue ischemia are based mainly on assessing blood flow and not blood perfusion [1, 5]. Palpation of pulses, ankle brachial index (ABI), duplex ultrasound, computed tomography angiography (CTA), magnetic resonance angiography (MRA), and some other methods evaluate blood flow exclusively. This is suitable for diagnosis and prognosis of a big artery disease (aortoiliac, above the knee). However, blood flow evaluation is less valuable in BTK or below the ankle (BTA) disease, especially when the toe pressure cannot be assessed [1]. Furthermore, even in cases with unaltered blood flow, patients could have ischemic wounds due to poor tissue perfusion. Several pathophysiological mechanisms come into play in diabetic patients. Diabetic polyneuropathy leads to sympathetic denervation, causing increased capillary permeability and opening of arterio-venous shunts [6, 7]. Thickening of the basal membrane causes arteriolar hyalinosis and impairs vasodilation [8].

Blood flow and tissue perfusion mismatch were discussed in the literature extensively [9, 10], however, there is still no valid method to evaluate perfusion. The main issue with blood perfusion measurement is a high variability among patients because of many confounding factors [1]: blood pressure, oxygen saturation, heart ejection fraction, peripheral spasm, environmental temperature, etc. It causes high variability in measurements even among the patients without PAD/SAD. The only partial exception is a ${\mathrm{TcPO}}_{2}$ measurement, with a negotiable 40 mmHg cut-off value [11, 12, 13], but it is inconvenient and time consuming.

Another issue regarding the evaluation of tissue perfusion is the lack of the reference standard. This turns into a validation problem of new techniques as tissue perfusion results should not be validated according to blood flow measurement. However, tissue perfusion results could be compared to the clinical outcome. Wound healing is a slow process, and that particular cohort of patients is very fragile. One-year cumulative amputation risk for amputation and death in a COMPASS trial was 23% and 9%, respectively [14]; yearly reintervention rate could be up to 30% [15]. That is why blood perfusion comparison to a long-lasting follow-up is questionable in this rapidly changing population.

A lot of new blood perfusion in tissues evaluating techniques have emerged recently, trying to prove their value: contrast-enhanced ultrasound [16, 17, 18]; MRI perfusion imaging [19, 20, 21]; hyperspectral imaging [22, 23]; laser doppler perfusion monitoring [12]; laser speckle contrast imaging [24, 25]; near-infrared spectroscopy [26, 27, 28, 29]; near-infrared fluorescence imaging with indocyanine green [30, 31]; spectrophotometry [32]; vascular optical tomography imaging [33]; photoacoustic imaging; micro-oxygen sensors [34] and some other emerging techniques. However scientific data is scarce and only two of the studies mentioned above have included more than 100 patients.

NIRS devices use several diodes of different wavelength, which has different penetration and absorption patterns by oxygenated and deoxygenated hemoglobin. Invos Oximeter (Somanetics/Medtronic) uses two diodes: 800 nm for oxygenated and deoxygenated hemoglobin and 760 nm for deoxygenated hemoglobin. The calculated measurement reflects tissue oxygenation. While tissue perfusion is not the same as tissue oxygenation, in this setting, where ischemic wound is associated with occluded BTK/BTA arteries, intraoperative changes of tissue oxygenation after revascularization reflect changes in tissue perfusion.

In our vascular center more than 800 lower limb endovascular interventions a year are performed. Having some prior not published expertise, which is entirely in line with the only published NIRS PAD clinical study by de Boezeman *et al*. [35], it was decided to investigate NIRS in a well-controlled clinical environment, as any variability in clinical cases (claudication vs. CLTI), intervention mode (open surgical vs. endovascular vs. BMT), anatomical site of lesion (aortoiliac vs. ATK vs. BTK), severe comorbidities, significantly affecting oxygen saturation, lead to very scattered results which are impossible to conclude in trials with a volume below ~1000 cases. An intraoperative measurement with a controlled environment as well as diminished interpatient variability in a small trial group could be a valuable proof of concept. If it fails, the possible benefit of NIRS measurement in a real-life scenario could be close to zero.

NIRS was evaluated in some wound healing clinical trials [36, 37, 38]. These trials showed oxygenated hemoglobin concentration differences in good versus poor healing wounds, however the wounds were not ischemic [37, 38], or only several of them ischemic [36]. This proves the ability to detect tissue oxygen changes using NIRS, however the results are scattered due to different ethiology of wounds and can hardly be translated to clinical practice.

Several systematic reviews were published summarizing possible benefits and shortcomings of NIRS in PAD evaluation [39, 40]. Despite the ability to detect PAD more precisely in some clinical scenarios, the main shortcoming was variability of the results which limited the clinical usage.

The aim of the study was to test if NIRS can detect the intraoperative increase of tissue oxygenation and if the elevation of NIRS ${\mathrm{rSO}}_{2}$ predicts wound healing in patients with CLTI and occlusion of below the knee arteries.

## 2. Materials and Methods

Vilnius Regional Biomedical Research Ethics Committee approved this study on Dec. 5, 2017, registration number 158200-17-981-482. The study was registered in clinicaltrials.gov on Apr. 2, 2019, registration number NCT03898869. Patients were included after obtaining informed consent.

The study was conducted in a tertiary non-university Vilniaus Miesto Klinikine Hospital, department of Vascular Surgery. The study was started Apr. 3, 2019, finished Sep. 7, 2020.

### 2.1 Patients

To avoid variability in measurements, strict inclusion and exclusion criteria were defined:

***Inclusions criteria***

• All comers PAD patients 55–95 years old;

• CLTI Rutherford V–VI;

• CTO below the knee;

• At least one artery below the knee was planned to be revascularized;

• No need for intervention in above the knee arteries.

***Exclusion criteria***

• Skin diseases preventing the use of NIRS;

• Life expectancy less than 12 months;

• Unavoidable amputation above the ankle;

• Systemic blood oxygen saturation below 85%.

All patients underwent standard clinical and laboratory investigation. The wounds were assessed according to WIfI classification [41]. The diagnosis of chronic total occlusion (CTO) was based on the results of angiography performed either at referring hospital or at the time of admission in our center.

### 2.2 Procedure and Measurements

Endovascular BTK/BTA revascularization procedure was performed by a single vascular surgeon using antegrade ipsilateral groin approach and retrograde pedal puncture when needed. All procedures were performed according to standard of practice (heparinization during the procedure, dual antiplatelet therapy for 3 months after the procedure). Short acting vasodilators were used only in the cases where distal puncture was needed. These medicines had no impact neither for initial measurements nor for final measurements.

All procedures were performed in an operating room equipped with Innova 4100, GE (Boston, Massachusetts, US). Postoperative images were anonymized and blindly evaluated by an independent interventional radiologist using Horos v3.3.6 (Annapolis, MD, USA). Technical angiographic success was scored 1 or 2, where 1—ranged from not successful recanalization to partialy successful recanalization, but without preservation of direct flow to pedal arch; 2—successful recanalization with full blood flow restoration through the pedal arch.

**Fig. 1.**
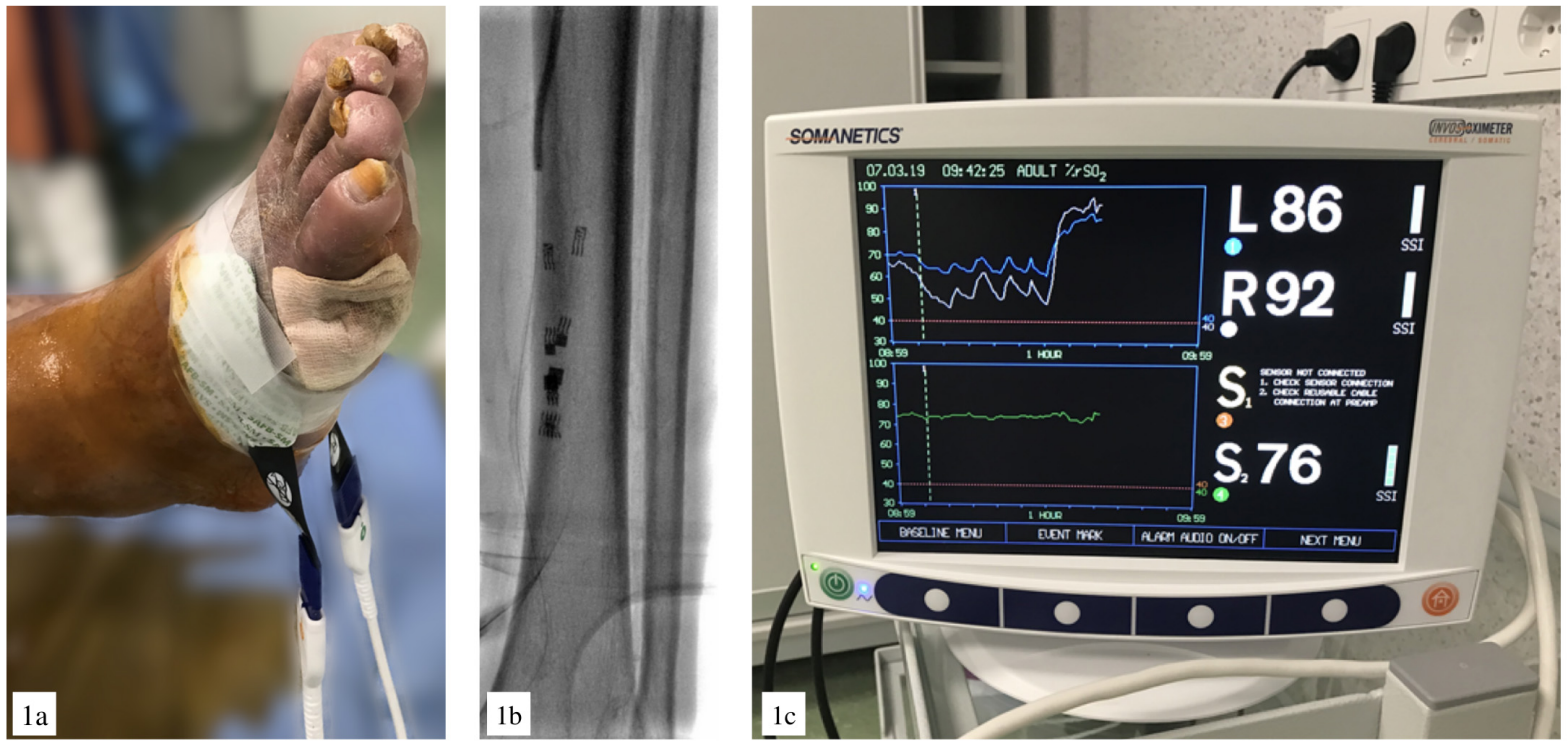
**NIRS application during revascularization procedure**. (a) Sensor placement near the wound. (b) X-ray view. (c) NIRS screen.

NIRS was measured using Invos Oximeter, Somanetics/Medtronic (Dublin, Ireland). Two probes were placed near the ischemic wound on muscular beds, and one reference probe was set on the pectoral muscle (Fig. 1a). Two sensors near the wound were placed on healthy skin, approximately 2–3 cm from ulcer margin. Because of sensors detecting oxygenation changes in ~2 cm depth, they were not placed over the tibial bone and over the bony prominences.

Despite the sensors were placed in the same region where revascularization was performed, they did not interfere with the X-ray view (Fig. 1b). The balloon inflation effect could be visualized on the NIRS screen (Fig. 1c).

After the procedure, NIRS data was downloaded and post processed using Excel v16.42, Microsoft (Redmond, WA, USA). ${\mathrm{rSO}}_{2}$ was recorded every 6 seconds. The mean of the first and the last 50 measurements of each sensor was calculated. The effect of revascularization was calculated using the formula below. The main goal was to zero the impact of fluctuations in systemic circulation using the reference sensor on the shoulder.

$$\begin{array}{c} \text{ Effect }=\left(\frac{\frac{\left(\mathrm{M1}\,\alpha +\mathrm{M2}\,\alpha \right)-\left(\mathrm{M1}\,\omega +\mathrm{M2}\,\omega \right)}{2}-\left(\mathrm{MR}\,\alpha -\mathrm{MR}\,\omega \right)}{\frac{\mathrm{M1}\,\alpha +\mathrm{M2}\,\alpha }{2}}-1\right)*100 \end{array}$$

$M_{1\,\alpha }$ — mean of the first 50 measurements on sensor 1 (before revascularization); $M_{2\,\alpha }$ — mean of the first 50 measurements on sensor 2 (before revascularization); $M_{1\,\omega }$ — mean of the last 50 measurements on sensor 1 (after revascularization); $M_{2\,\omega }$ — mean of the last 50 measurements on sensor 2 (after revascularization); $M_{\text{R}\,\alpha }$ — mean of the first 50 measurements on reference sensor (before revascularization); $M_{\text{R}\,\omega }$ — mean of the last 50 measurements on reference sensor (after revascularization).

A right shoulder was used for baseline sensor. The other leg is usually altered by the same disease. The blood perfusion in arms might be altered by prior dialysis access surgery, as end stage renal disease is quite abundant in this patient cohort. That is why a shoulder was chosen as a baseline place.

Vital signs and oxygen saturation on the index finger were monitored using B40 Patient Monitor, GE (Boston, Massachusetts, USA).

The final anonymized angiographic results from all 30 subjects were evaluated by an independent radiologist and patients were assigned into two groups. The first group—suboptimal angiographic result, when the blood flow to the pedal arch was not restored. The second group — good angiographic result with blood flow restoration to the pedal arch.

ABI was measured using Dopplex® Ankle Brachial Pressure Index Kit with EZ8 8MHz Probe, Huntleigh (Cardiff, Wales, UK).

### 2.3 Follow-Up

A follow-up visit was set up 30 days after the discharge for each patient. Wounds were reassessed using WIfI classification by the same vascular surgeon. Wound healing after one month was evaluated using WIfI classification. Because of small number of patients, scattered initial wound characteristics and different healing patterns, the second type of grading was used, based on healing pattern as 1 or 2, where 1—no improvement or slight improvement; 2—wound is healed or is healing rapidly and there is tendency for complete heal in the near future.

### 2.4 Statistical Analysis

Statistics were performed using SPSS 26.0 (IBM, Armonk, NY, USA) and power calculations were performed using GPower version 3.1 (HHU, Dusseldorf, Germany). The Gaussian distribution and homogeneity of variance of the data were confirmed using Shapiro-Wilk and Levene tests. Data are presented as the mean ± SD for normal distributed values, otherwise median and interquartile range (25 and 75 percentile) (IQR). Outliers were defined as more than mean ± 3SD and excluded from further analysis. Statistical significance was assessed using Student’s *t*-test for normal distributed data, the Wilcoxon signed rank test for continuous non normal distributed variables and Fisher’s exact test for categorical variables. The difference between samples was considered statistically significant if the *p* value was less than 0.05.

### 2.5 Power Analysis

There was no preliminary data we could use to perform power analysis before the trial. The sample size was chosen to be twice as big as the cohort of the only previous clinical trial dealing with intraoperative endovascular NIRS measurement [35] and the only trial dealing with intraoperative open surgery NIRS measurement. The sample size was also based on other blood perfusion tests (hyperspectral imaging, laser doppler perfusion monitoring, near-infrared fluorescence imaging with indocyanine green, micro-oxygen sensors, etc.) listed previously.

The post-hoc Power analysis was performed after calculating the results. The sample size for matched pairs *T*-test with α error probability of 0.05 and Power (1 - β error probability) of 0.95 is 23 subjects to detect difference between preoperative and postoperative results. The sample size for independent groups *T*-test with α error probability of 0.05 and Power (1 - β error probability) of 0.8 is 38 subjects to detect difference between two patient groups based on postoperative results and different clinical outcomes.

## 3. Results

30 patients were enrolled into the study. There were 17 males (57%), the mean age of the patients was 74.7 ± 11.2 years. 16 patients (53%) had diabetes mellitus, 10 (33%) had end-stage renal disease (Table 1). All patients had chronic total occlusion below the knee and the ischemia was classified as Rutherford category V (Table 2). NIRS ${\mathrm{rSO}}_{2}$ measurements and other intervention data that were collected during the procedure are depicted in Tables 2,3.

**Table 1.** **Demographic data**.

Variables	No. (%) or Mean ± SD
Study patients	30
Age, years	74.7 ± 11.2
Male	17 (57%)
Caucasian	30 (100%)
Diabetes mellitus	16 (53%)
End stage renal disease	10 (33%)
Hypertension	24 (80%)
Coronary artery disease	21 (70%)

**Table 2.** **Initial clinical data**.

Variables	No. (%) or Mean ± SD
CLTI, Rutherford V	30 (100%)
CTO below the knee	30 (100%)
Concomitant SFA disease, requiring treatment	1 (3%)
Previous open surgery on index leg	3 (10%)
Previous endovascular intervention on index leg	13 (43%)
Previous minor amputations on index leg	6 (20%)
Elevated CRP on admission	12 (40%)
Increased WBC on admission	10 (33%)
WIfI, W2	14 (46%)
WifI, I2	15 (50%)
WifI, fI1	12 (40%)
ABI (20 patients)	0.6 [0.21]
CTO, intended to treat P3	1 (3%)
CTO, intended to treat distal to popliteal artery	30 (100%)
BP, systolic at the beginning of procedure	154 ± 19.4
Oxygen saturation, %	94 ± 2.5

CLTI, chronic limb threatening ischemia; CTO, chronic total occlusion; SFA, superficial femoral artery; CRP, C reactive protein; WBC, white blood cells; WIfI, the classification system proposed by the Society for Vascular Surgery (W, Wound; I, Ischaemia; fI, foot Infection); ABI, ankle brachial index; BP, blood pressure.

**Table 3.** **Intervention data**.

Sensor	NIRS ${\mathrm{rSO}}_{2}$ before the reperfusion, Mean ± SD	NIRS ${\mathrm{rSO}}_{2}$ after the reperfusion, Mean ± SD	*p* value
Sensor 1	58.0 ± 12.7	66.7 ± 11.6	0.001
Sensor 2	57.6 ± 12.7	67.1 ± 14.0	<0.001
Reference sensor	67.7 ± 11.3	63.1 ± 12.0	0.001

CTO, chronic total occlusion; NIRS, near infrared spectroscopy.

Statistically significant NIRS ${\mathrm{rSO}}_{2}$ increase (Table 3) was observed on sensors near the wound after the reperfusion (paired samples *T*-test, *p* = 0.001). Statistically significant NIRS ${\mathrm{rSO}}_{2}$ decrease during the procedure on reference sensor during the procedure (paired samples *T*-test, *p* = 0.001).

Independent anonymous evaluation of revascularization success was performed. The success was rated as suboptimal in 12 (40%) cases (group 1) and optimal in 18 (60%) cases (group 2).

### Follow-Up

3 patients (10%) died during the first 30 days, therefore the follow up included 27 patients. Wound healing after 30 days was evaluated as poor in 9 patients (30%) and good in 18 patients (70%). Follow-up ABI median was 0.7 [0.2].

Patients with different angiographic revascularization success were stratified by their clinical outcome (the course of wound healing) (Table 4). There was no relationship between the angiographic success and wound healing categories (Fisher exact test, *p* = 0.683). Initial and final ABI, initial and final NIRS ${\mathrm{rSO}}_{2}$ values, current comorbidities did not correlate with wound healing also.

**Table 4.** **Comparison of angiographic results and wound healing**.

	Suboptimal angiographic result	Optimal angiographic result
Poor wound healing	4	5
Good wound healing	6	12

Fisher exact test used, *p* = 0.683

Comparing good wound healing group vs. poor wound healing group intraoperative NIRS ${\mathrm{rSO}}_{2}$ increase difference was statistically significant, *p* = 0.017 (Table 5). Three statistical outliners were excluded from calculations.

**Table 5.** **Comparison of NIRS ${\mathrm{rSO}}_{2}$ change in different wound healing groups**.

Poor wound healing		Good wound healing		
NIRS ${\mathrm{rSO}}_{2}$ change after the revascularization, Mean ± SD	No. of patients	NIRS ${\mathrm{rSO}}_{2}$ change after the revascularization, Mean ± SD	No. of patients	*p* value
13.6 ± 3.3	6	27.2 ± 25.2	18	0.017

Student’s *t*-test used for comparison. NIRS, near infrared spectroscopy.

## 4. Discussion

NIRS is a non-invasive method which is not harmful to tissue even if applied for a longer period of time [42]. In contrast to other methods measuring tissue perfusion such as ${\mathrm{TcPO}}_{2}$, NIRS does not require skin to be heated prior to measuring tissue perfusion and it is not so operator dependant as hyperspectral imaging [1, 22]. Compared to micro-oxygen sensors (MOXY), NIRS appears to be a less expensive, non invasive and easier to perform technique [34]. Also, application of NIRS does not require additional contrast media and standartized protocol as it must be done while using 2D perfusion angiography [43].

INVOS™ Cerebral/Somatic Oximetry device, which was used in this study, is not the most optimal NIRS device for detecting peripheral tissue perfusion. It was chosen because it has a CE Mark and is broadly available in clinical practice.

To the best of our knowledge this is the second study in the world evaluating intraoperative NIRS results. The first study, conducted by Boezeman *et al*. [35], showed no NIRS ${\mathrm{rSO}}_{2}$ increase after the revascularization. However, that study included only 14 patients, 43% of them were without gangrene, 79% of the lesions were above the knee, no data was obtained comparing stenosis versus CTO.

Our experience evaluating blood perfusion using NIRS outside this study patients replicated the findings published by Boezeman *et al*. [35]. Small intraprocedural NIRS ${\mathrm{rSO}}_{2}$ increase is influenced by numerous factors, such as spasm following introducer sheath insertion, blood pressure fluctuations, patient hyperventilation, etc. The most significant NIRS ${\mathrm{rSO}}_{2}$ increase was observed after the direct blood flow restoration to the target area. Therefore, an assumption was made that if NIRS measurement could prove its value, it would do it in specifically controlled environment.

A significant increase in NIRS ${\mathrm{rSO}}_{2}$ was demonstrated after restoration of blood perfusion in the current study. This lets us think that NIRS can be used for intraoperative blood perfusion measuring in patients with CTO and below the knee occlusion. Moreover, a higher increase in NIRS ${\mathrm{rSO}}_{2}$ after revascularization was associated with better wound healing. In this setting, the increase in NIRS ${\mathrm{rSO}}_{2}$ served as prognostic marker of wound healing and even outperformed the predictive potential of independent angiographic evaluation of revascularization.

The first approach to test tissue perfusion during described revascularization procedure proved to be successful. However, it is a far cry from everyday day use in clinical practice. The next step could be validating this technology comparing with ${\mathrm{TcPO}}_{2}$ measurements before and after the procedure, repeating NIRS and ${\mathrm{TcPO}}_{2}$ on 30-day follow-up visit.

## 5. Limitations of Technology

All calculations were made after postprocessing of the data, where the baseline was connected to a reference sensor value. As baseline data is changing during the procedure, it is important to note that the current Invos Somanetics device uses a different formula and the results shown on screen are not straightforward.

The price of each single-use sensor is equal to the price of a PTA balloon. Using three sensors per procedure on a routine basis could increase the average procedure cost. On the other hand, even the small improvement in treatment strategy may contribute to huge savings in ulcer treatment. This was demonstrated by Weingarten *et al*. [44] with an earlier detection of wound healing failure using NIRS allowing to save more than 12,000 USD per patient.

The impact of severe local inflammation, lung and heart diseases affecting oxygen saturation, alter the measurements and might limit the usage of this technology.

## 6. Limitations of the Study

Despite rigorous inclusion criteria, an additional one could have been included. Severe local inflammation impacts the measurement, so fI 2 and 3, according to WIfI classification, could have been excluded.

The only validated tissue perfusion measurement ${\mathrm{TcPO}}_{2}$ before and after the procedure could have been included in the comparison. We have not included it, as the primary idea was to put NIRS sensors as close to the wound as possible and ${\mathrm{TcPO}}_{2}$ measurement has more defined areas. However, a future study comparing ${\mathrm{TcPO}}_{2}$ and NIRS ${\mathrm{rSO}}_{2}$ with a different patient group, giving more attention to the wound characteristics and location, could be planned. Some studies have compared NIRS and ${\mathrm{TcPO}}_{2}$ [45] with promising results.

## 7. Future Perspectives

We see a potential of NIRS measurements in evaluating ischemic tissue perfusion. Possibility to revascularize one, two, or three BTK arteries is a nice option enabled by endovascular technique. However, a patient with CLTI is usually very fragile and the need to shorten the intervention is widely expressed. The ability to monitor tissue perfusion near the wound during the procedure and stop the procedure once required increase is achieved would be extremely valuable. Currently there are no devices certified for detection of tissue perfusion changes during revascularization. The need to postprocess data is a clear barrier for every day clinical use of existing devices. That is why, further adoption of this technology is limited to manufacturers of existing devices.

Positive results of this study set background for further investigation. Future studies are needed to assess the ability of NIRS to predict wound healing, minor and major amputation in larger patient cohorts.

## 8. Conclusions

NIRS is feasible method for detecting tissue perfusion changes during endovascular revascularization of BTK and BTA arteries. However, a dedicated device with a modified measurement technique is needed.
